# The Cost of Bad Timing: How Phenology and Frequency Determine Agricultural Flood Risk

**DOI:** 10.1007/s11269-026-04801-1

**Published:** 2026-06-15

**Authors:** Shokhrukh-Mirzo Jalilov, Robert Maltsbarger, Haluk Gedikoglu

**Affiliations:** Division of Applied Social Sciences, University of Missouri, Columbia, MO USA

**Keywords:** Agricultural flood risk, Agricultural loss, Expected annual damage, Crop phenology, Missouri river, Chariton county

## Abstract

Accurate valuation of flood risk is fundamental to efficient resource allocation, insurance pricing, and public investment in agriculture. Standard economic models, which often link asset damage directly to hazard magnitude, fail to capture the unique vulnerability of agricultural capital—where the value of standing crops is contingent upon phenological stage. This study develops a capital valuation framework to deconstruct the economic burden of flooding on cropland, demonstrating that the timing of a flood is a primary determinant of financial loss, often outweighing the role of physical flood magnitude. We model agricultural flood risk as expected annual damage (EAD) to crop capital, integrating hydrologic frequency analysis for a U.S. Midwest County with a phenologically-explicit damage function. This function disaggregates risk into two components: a Flood Hazard Index (FHI), quantifying flood intensity and duration across return periods, and a Flood Susceptibility Index (FSI), representing the time-sensitive depreciation rate of crop capital at different growth stages. Probability-weighted losses are summed across all flood scenarios to derive total EAD. Results reveal that the distribution of losses is heavily skewed toward high-probability, low-severity events. The 2-year and 25-year floods collectively account for approximately 45% of total EAD, despite extreme (≥ 100-year) events generating substantially larger per-event losses. Frequent floods impose the highest economic cost not because of peak discharge, but because their high likelihood of coinciding with phenologically sensitive periods is compounded by longer inundation durations—a “double liability” where occurrence probability and capital impairment duration are simultaneously maximized. Conversely, low-probability, high-severity events tend to occur outside the growing season, leaving lower-value capital exposed. These findings invert conventional risk models and carry significant implications for crop insurance design, flood mitigation investment, and agricultural capital management under climate volatility.

**Supplementary Information:**

The online version contains supplementary material available at 10.1007/s11269-026-04801-1.

## Introduction

### Economic Mispricing of Agricultural Flood Risk

Flood impacts are projected to intensify globally over the coming decades, driven by the increasing frequency of extreme weather events and continued population growth in flood-prone areas (Qiang et al. 2017; Merz et al. 2021). These impacts manifest through direct physical damage to infrastructure and assets, indirect economic losses resulting from reduced productivity and business interruptions, and, in severe cases, loss of human life. Flood damage assessments have largely concentrated on residential, infrastructure, and industrial sectors, with comparatively limited focus on agriculture (Merz et al. 2009; Brémond et al. 2013). Yet, flooding can severely impact agricultural production, highlighting the need for robust methodologies to evaluate flood-related agricultural damages (Shrestha et al. 2018). In the United States alone, flooding has caused substantial damages, accounting for approximately 7.4% of total economic losses associated with natural disasters (Abegaz et al. 2024).

Flood risk management has been extensively examined across a wide range of disciplines, including civil engineering, economics, sociology, cultural studies, and psychology (Vozinaki et al. 2015). Flood risk analysis and damage assessment constitute a long-standing theme in *Water Resources Management*, encompassing systematic reviews of damage estimation methods with attention to data-scarce contexts (Romali 2025), hydrological modelling of mitigation strategies such as reservoir-based flood control under climate change (Ikemoto et al. 2023), automated DEM correction for urban inundation modelling (Khalid et al. 2025), decision-support frameworks for flood impacts on water supply systems (Barbetta et al. 2022), flood susceptibility mapping using data-driven approaches (Akay 2024), and quantification of flood control benefits under uncertainty in flood peak and frequency (Zhang et al. 2025). Despite this broad scholarly engagement, flooding continues to represent the most frequent and damaging natural hazard worldwide on an annual basis, indicating that current understanding remains incomplete, fragmented, and methodologically limited. Therefore, robust and accurate assessment of flood impacts is essential for effective flood risk management, impact mitigation, adaptive planning, and the evaluation of the benefits of forecasting and early warning systems (Redondo-Tilano et al. 2025). To date, no comprehensive damage assessment framework has successfully integrated the full spectrum of flood characteristics—such as seasonality, water depth, flow velocity, flood duration, and sediment deposition—within a single damage estimation model (Merz et al. 2010; Kreibich et al. 2017; Mohor et al. 2018). Nonetheless, prior studies have attempted to improve damage estimation by incorporating subsets of these influencing parameters into flood damage functions (Vozinaki et al. 2015).

Flood risk valuation underpins agricultural insurance design, floodplain regulation, and public investment in mitigation infrastructure. In economic terms, flood risk is commonly represented by expected annual damage (EAD), defined as the probability-weighted sum of losses across events of varying magnitude (Foudi et al. 2015; Wang et al. 2023). While this framework is well established, its application to agriculture remains incomplete. Most flood damage assessments implicitly assume that damages are primarily a function of flood magnitude and spatial exposure—an assumption appropriate for infrastructure assets with relatively stable value over time but problematic for biological production systems (Meyer et al. 2013).

In agricultural systems, the value at risk is embodied in standing crops whose economic value and vulnerability vary systematically throughout the growing season (USDA 2016). Consequently, floods of identical magnitude can generate substantially different losses depending on their timing relative to crop development, with seasonal floods coinciding with sensitive growth stages contributing disproportionately to expected damages despite moderate severity. Empirical evidence shows that ignoring this temporal variation biases flood damage estimates and distorts risk signals for farmers and policymakers (Wing et al. 2020; Davenport et al. 2021). However, most agricultural flood risk assessments still rely on static or season-averaged damage functions that obscure these interactions (Förster et al. 2008; Thieken et al. 2017). This study addresses this gap by explicitly incorporating temporal variation in both flood hazard and crop vulnerability within an expected-value framework.

A dominant institutional framework shaping the economic valuation of agricultural flood risk in the United States is the U.S. Army Corps of Engineers (USACE) National Economic Development (NED) methodology for agricultural flood damage estimation (USACE 1987). The USACE approach operationalizes flood risk through EAD calculations, combining flood frequency information with crop-specific damage functions, cropping patterns, yields, and market prices. Flood impacts are evaluated using either continuous-record simulations of historical events or frequency-based analyses in which damages from synthetic flood events are weighted by their annual exceedance probabilities (USACE 1987).

Within this framework, crop losses are represented using stage–damage and seasonal crop loss functions that relate flood occurrence to percentage yield loss depending on the timing of inundation within the crop production cycle. Seasonality is explicitly acknowledged through broad temporal categories corresponding to planting, growing, and harvest periods (USACE 1987). Flood duration is recognized as an important driver of crop damage; however, it is generally incorporated implicitly through elevation zoning and mean daily discharge assumptions rather than treated as an explicit, continuous determinant of yield loss. As a result, damages are largely evaluated at representative flood stages or seasonal averages rather than derived from the full temporal structure of flood events (USACE 1987).

While the USACE methodology provides a consistent basis for large-scale project appraisal, its reliance on aggregated seasonal damage functions and peak-based hazard representations can misrepresent agricultural flood risk. In particular, moderate but recurrent events occurring during sensitive growth stages—and driven more by duration than peak magnitude—may be systematically underrepresented in expected annual damage (EAD) estimates. This aggregation weakens risk signals relevant for land-use planning, insurance pricing, and investment decisions by masking interactions between hazard occurrence and crop vulnerability.

The agricultural economics literature increasingly recognizes weather-related production risk and its interaction with insurance institutions. Sherrick et al. (2004) and Glauber (2012) document the evolution of federal crop insurance and behavioral responses to risk. Recent studies examine adaptation to climate risk (Annan and Schlenker 2015), improved actuarial rating using weather data (Liu and Ramsey 2023), and agronomic mitigation of excess moisture losses (Aglasan et al. 2024). However, most analyses remain focused on seasonal or annual aggregates and do not explicitly resolve how intra-seasonal hazard timing interacts with crop phenology to determine expected damages, which is the focus of this study.

The proposed method builds on the economic logic of the USACE framework while extending it by explicitly resolving the temporal dimensions of flood hazard and crop susceptibility. By modeling flood occurrence probabilistically at the monthly scale and incorporating flood duration as a core damage determinant, the approach provides a more process-aware representation of agricultural flood risk. In doing so, it addresses key sources of economic mispricing embedded in traditional valuation frameworks and enables more accurate estimation of expected losses in biologically dynamic production systems.

### Regional Context and Study Area: Chariton County, Missouri

Flood hazards such as riverine floods and storm surges are typically localized events. As a result, their impacts tend to be spatially concentrated and are unlikely to generate significant macroeconomic effects at the national level (Ludwig and Brautzsch 2002). Consequently, flood risk assessment and mitigation are most effectively undertaken at finer geographic scales where exposure, vulnerability, and damages are realized. This study therefore adopts a sub-basin, county-level perspective and focuses on Chariton County, Missouri—a predominantly agricultural county situated along the Missouri River within the Lower Missouri River Basin. The basin provides critical hydrologic context, as it is characterized by pronounced seasonal flood clustering driven by spring precipitation and upstream snowmelt, which elevates flood probabilities during the primary crop growing season. This confluence of hydrologic forcing, extensive agricultural land use, and proximity to a major regulated river system renders Chariton County particularly vulnerable to flood-induced agricultural losses (USGS 2018).

### Study Objectives

The objective of this study is to provide a probabilistically consistent valuation of agricultural flood risk that explicitly accounts for the timing of floods relative to crop development. Rather than testing formal hypotheses or estimating efficiency gains from specific interventions, the analysis focuses on risk characterization and decomposition within an expected-value framework consistent with established economic guidance (FEMA 2023).

This study advances beyond previous agricultural flood damage assessments through three methodological innovations. First, it integrates flood duration and occurrence probability at monthly resolution, replacing seasonal averages with temporally explicit hazard characterization. Second, it employs phenology-specific vulnerability functions linked to empirical crop tolerance thresholds, rather than static depth-damage curves that assume uniform susceptibility across growth stages. Third, it decomposes EAD across both frequency and seasonal dimensions, enabling identification of when and how often risk materializes. While previous studies have incorporated subsets of these elements (USACE 1987; Vozinaki et al. 2015), no prior work has integrated flood persistence, monthly timing, and event probability within a unified expected-value framework calibrated to crop physiological response.

The novelty of this study is the integration of flood frequency analysis and crop phenological vulnerability into a single probabilistic damage framework, overcoming limitations of conventional EAD models that use static seasonal damage functions and peak-discharge hazard metrics. The framework combines the Flood Hazard Index (FHI), representing flood intensity and duration across return periods, with the Flood Susceptibility Index (FSI), capturing crop vulnerability by growth stage.

Although demonstrated for Chariton County, the framework is transferable to other regions. Its three core inputs — gauge-based flood frequency distributions, phenologically explicit vulnerability indices, and spatially explicit crop exposure — are nationally available through USGS streamflow networks, USDA Cropland Data Layers, and FEMA/DSWE flood mapping. Application to other regions requires re-estimating conditional monthly flood probabilities from local gauge records and adjusting FSI values to reflect local crop calendars; the FHI/FSI structure itself can accommodate different crop systems, climate regimes, and seasonal flood timing without structural modification.

## Methodology of Agricultural Flood Risk Assessment

### Conceptual Framework

**Fig. 1:**
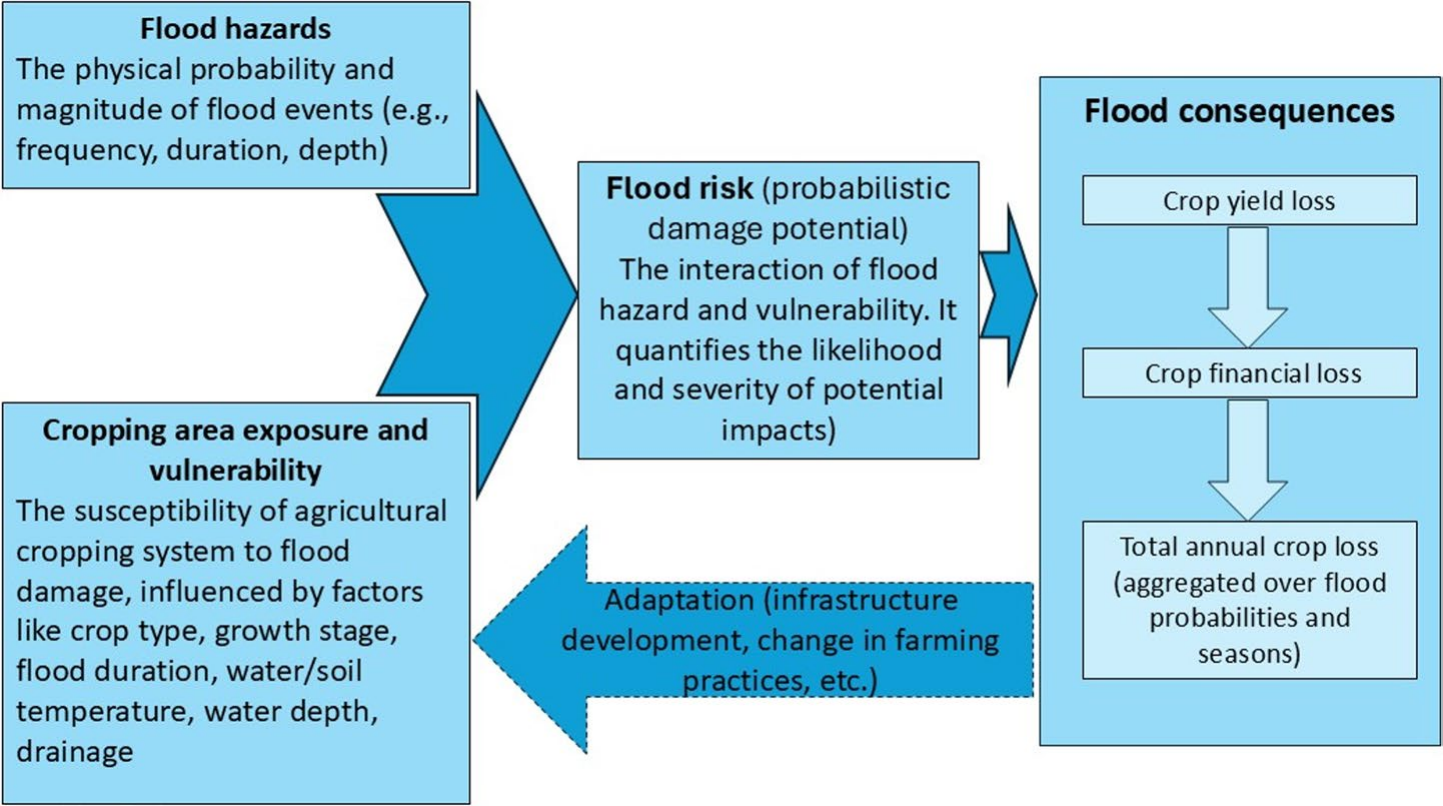
Conceptual framework for estimating expected annual damage (EAD) from flooding

Figure 1 presents the conceptual framework underlying this agricultural flood loss analysis, structured around the standard risk paradigm where risk emerges from the interaction of hazard, exposure and vulnerability (Penning-Rowsell et al. 2005; Foudi and Osés-Eraso 2014; Foudi et al. 2015; Huang 2018; Efraimidou and Spiliotis 2024). Flood hazards are characterized by the probability, magnitude, timing, and duration of inundation events based on historical discharge data. These hazards act as external drivers of agricultural system outcomes. Cropping area exposure and vulnerability characterize the susceptibility of agricultural production systems to flood damage. Exposure refers to the spatial intersection of cultivated lands with flood-prone area, determining which crops and acreages are subject to potential inundation. Vulnerability encompasses the biophysical and management factors that govern damage severity given exposure, including crop variety, phenological growth stage at the time of flooding, flood duration tolerance, soil drainage characteristics, water temperature, and inundation depth. Vulnerability is inherently dynamic, varying across the growing season as crops progress through developmental stages with differential flood tolerance.

Flood risk arises from the interaction between hazard and vulnerability, integrating flood probability distributions with stage-specific crop vulnerability functions. Critically, identical flood events produce vastly different agricultural impacts depending on timing: a spring flood during planting differs fundamentally from a summer flood during grain fill. Crop vulnerability is operationalized through the Flood Susceptibility Index (FSI), a time-dependent depreciation rate applied to standing crop value. Yield losses are expressed as fractional reductions from expected non-flood yields, converted to financial losses via commodity prices, and aggregated spatially and temporally across all flood scenarios (magnitude × timing × duration) to derive EAD.

While this study does not model adaptive responses, the conceptual framework (Fig. 1) incorporates an adaptation feedback loop recognizing that structural interventions, land-use adjustments, and farm-level responses (e.g., flood-tolerant varieties, altered planting dates, crop insurance) can reduce exposure or vulnerability — and that quantified risk estimates like EAD are the necessary inputs to evaluating such measures.

### Hydrologic Data and Analysis

#### Background Information

Shrestha et al. (2016) emphasized that historical records of flood hazards, associated damages and their interrelationships are essential for developing robust methodological approaches and for validating modelled outcomes. Thus, daily river discharge data were obtained from the U.S. Geological Survey (USGS) Current Water Data for the Nation (https://waterdata.usgs.gov/nwis/rt) for the Waverly stream gauge on the Missouri River (USGS station 06895500: Missouri River at Waverly, Missouri), which is located upstream of Chariton County and defines the hydrologic boundary of the study area. The dataset covers the period from 1 October 1928 to 30 September 2025, providing a 97-year historical record suitable for flood frequency and probabilistic analysis. The discharge series exhibits strong positive skewness and heavy tails, reflecting infrequent but extreme flood events typical of large, regulated river systems, thereby motivating a probabilistic flood risk framework.

**Fig. 2:**
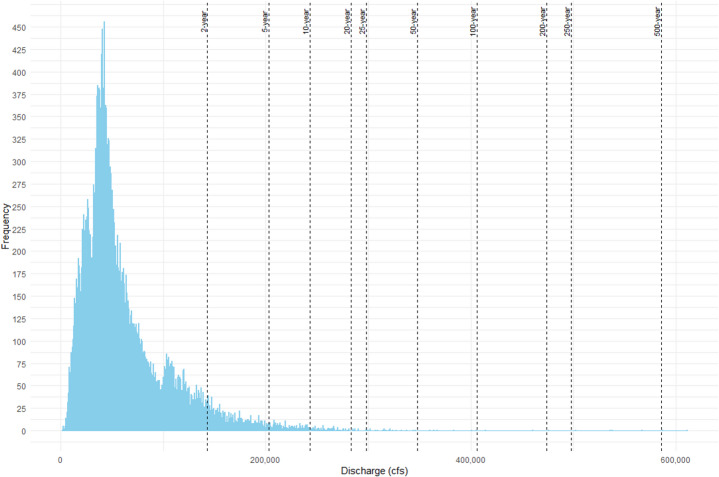
Missouri river discharge frequency at Waverly gauge and corresponding USACE return-period peak flows for risk assessment

Figure 2 presents the frequency distribution of daily discharge at the Waverly stream gauge. The histogram reveals a strongly right-skewed distribution characteristic of river flow regimes, where the vast majority of daily discharge observations are concentrated at lower to moderate flow levels, predominantly below 100,000 cfs, while a long tail extends toward substantially higher discharge values associated with rare flood events. As discharge increases beyond 100,000 cfs, the frequency of occurrence decreases rapidly, demonstrating the relative rarity of high-flow events. Overlaid on the frequency distribution are vertical dashed lines indicating peak discharge levels corresponding to standard annual exceedance probabilities (AEPs) developed by the U.S. Army Corps of Engineers (USACE 2023). These thresholds represent AEPs of 50%, 20%, 10%, 5%, 4%, 2%, 1%, 0.5%, 0.4%, and 0.2%, equivalent to 2-, 5-, 10-, 20-, 25-, 50-, 100-, 200-, 250-, and 500-year flood return periods, respectively.

Table 1 illustrates peak flow associated with each of AEPS. These probabilistic flood frequency estimates are derived from statistical analysis of historical flood peaks and provide standardized benchmarks for characterizing flood hazard magnitude and likelihood (USACE 2023).

**Table 1:** Flood frequency discharge thresholds and annual exceedance probabilities, Missouri River at Waverly

AEP (%)	50	20	10	5	4	2	1	0.5	0.4	0.2
Return period	2-year	5-year	10-year	20-year	25-year	50-year	100-year	200-year	250-year	500-year
Flow (cfs)	143,000	203,000	243,000	283,000	298,000	348,000	406,000	474,000	498,000	586,000

Source: USACE (2023)

#### Temporal Distribution of Flood Probabilities

To characterize seasonal flood patterns across return periods, annual flood probabilities were decomposed into monthly components. For each annual exceedance probability (AEP) threshold, conditional probabilities were calculated to represent the likelihood that a flood occurs in each month given that the annual maximum discharge exceeds the threshold. This was done by identifying all exceedance years, extracting the month of occurrence, and computing the frequency distribution across months. The resulting probabilities sum to one for each return period. Monthly AEPs were then obtained by multiplying these conditional probabilities by the corresponding annual AEP.

This temporal decomposition enables crop-stage-specific damage estimation by linking flood occurrence with crop phenology. Because crop vulnerability varies across growth stages, the same flood magnitude can produce different impacts depending on when it occurs. Flood timing is therefore integrated with crop development stages to define distinct hazard–impact scenarios. The analysis assumes that the historical seasonal distribution of floods within the regulated Missouri River system is representative of current conditions. This reflects the managed hydrologic regime under which agricultural production occurs. Potential future changes in climate or river operations are not considered, as the objective is to estimate expected impacts under observed conditions. Detailed interpretation of seasonal patterns by return period is provided in the Supplementary Information (SI).

#### Flood Duration Analysis

While flood magnitude and timing determine initial exposure, duration critically influences damage severity. Flooding substantially reduces oxygen availability in soils, promoting anaerobic microbial activity that adversely affects plant physiological processes (Rohilla et al. 2020; Loreti and Perata 2020) and overall crop productivity (Mishra et al. 2015). Oxygen deficiency under prolonged inundation constrains root development and can result in root mortality (Aslam et al. 2023; Timmerman et al. 2018). In addition, flooding disrupts the uptake and transport of water and nutrients, leading to suppressed plant growth and reduced field-level production (Zheng et al. 2009; Pampana et al. 2016). To characterize duration patterns across return periods and seasons, we analyzed exceedance duration at each USACE AEP threshold throughout the 97-year record.

We implemented a flood event identification algorithm to characterize discrete flood events rather than simply counting exceedance days. A flood event was defined as a continuous period during which daily discharge remained above a specified threshold, with a maximum gap tolerance of 3 days. This gap parameter accounts for multi-peak hydrographs characteristic of large river systems, where brief recessions do not represent true flood termination. For each identified event, we extracted start date, end date, peak discharge, duration (days), and month of occurrence. This event-based approach provides realistic representation of how floods actually occur and affect agricultural operations.

To characterize seasonal variation in flood duration, we calculated monthly exceedance days—the number of days within each month that discharge exceeded each threshold. This analysis was conducted by filtering daily discharge data, grouping exceedance days by year and month, and compiling results across the full record. The resulting 1,164 month-level observations (97 years × 12 months) for each AEP threshold enable robust statistical characterization of seasonal duration patterns aligned with crop growth stage frameworks. Identified flood events were subsequently classified into four flood durations categories which are agronomically meaningful classes based on crop physiological response thresholds:

- 1–3 days: Brief inundation causing minimal to moderate stress with recovery potential.
- 4–7 days: Extended inundation causing severe stress and significant yield reduction (30–60% loss).
- 8–14 days: Prolonged inundation resulting in major yield loss (60–90%) or complete stand loss.
- > 15 days: Extreme prolonged inundation causing complete crop loss across major Midwest row crops.

Detailed interpretation of these duration thresholds is provided in the Supplementary Information (SI).

### Integration with Agricultural Vulnerability Assessment

#### Flood Hazard Index (FHI)

The monthly flood probability distributions derived from the discharge analysis form the hydrological basis for constructing the Flood Hazard Index (FHI), which is designed to capture the combined effects of flood intensity and flood duration on agricultural systems (Table 2). The FHI is a synthetic, dimensionless indicator that represents the relative severity of crop damage arising from the interaction between flow magnitude (expressed as monthly flood intensity fractions) and the persistence of inundation (expressed as flood duration categories).

Table 2 presents the full FHI matrix. Values increase monotonically with both intensity and duration, reaching unity for prolonged (≥ 15 days) or very high-intensity events. Full details of matrix construction are provided in the SI.

**Table 2:** Ordinal Flood Hazard Index (FHI) classification based on flood intensity and duration

Return Period	Month	Flood probability	Flood duration	Monthly discharge fraction	FHI
2-year	Mar	0.033	> 15	0.07	1.0
	Apr	0.066	> 15	0.13	1.0
	May	0.098	> 15	0.20	1.0
	Jun	0.172	> 15	0.34	1.0
	Jul	0.057	> 15	0.11	1.0
	Aug	0.008	> 15	0.02	1.0
	Sep	0.025	> 15	0.05	1.0
	Oct	0.033	> 15	0.07	1.0
	Nov	0.008	4–7	0.02	0.1
5-year	Mar	0.012	> 15	0.06	1.0
	Apr	0.030	> 15	0.15	1.0
	May	0.024	8–14	0.12	0.8
	Jun	0.073	> 15	0.36	1.0
	Jul	0.030	> 15	0.15	1.0
	Sep	0.006	1–3	0.03	0.1
	Oct	0.024	4–7	0.12	0.4
10-year	Mar	0.006	4–7	0.06	0.4
	Apr	0.013	8–14	0.13	0.8
	May	0.019	8–14	0.19	0.8
	Jun	0.031	8–14	0.31	1.0
	Jul	0.025	> 15	0.25	1.0
	Oct	0.006	1–3	0.06	0.1
20-year	Apr	0.013	4–7	0.25	0.4
	May	0.013	1–3	0.25	0.1
	Jun	0.013	4–7	0.25	0.4
	Jul	0.013	8–14	0.25	0.8
25-year	Apr	0.013	4–7	0.33	0.7
	Jun	0.013	4–7	0.33	0.7
	Jul	0.013	8–14	0.33	1.0
50-year	Apr	0.007	1–3	0.33	0.3
	Jul	0.013	8–14	0.67	1.0
100-year	Jul	0.010	4–7	1.00	1.0
200-year	Jul	0.005	4–7	1.00	1.0
250-year	Jul	0.004	4–7	1.00	1.0
500-year	Jul	0.002	1–3	1.00	0.8

#### Flood Susceptibility Index (FSI)

Table 3 presents the monthly Flood Susceptibility Index (FSI) for corn, soybeans, and winter wheat, designed to represent the relative sensitivity of crops to flooding across their phenological cycles. The FSI is a dimensionless indicator ranging from 0 (no susceptibility) to 1 (maximum susceptibility) and reflects the likelihood that flooding occurring during a given month would cause meaningful crop damage. The index was developed based on the phenology of three dominant Midwestern U.S. crops—corn, soybeans, and winter wheat—using evidence from agronomic literature and extension guidance. Susceptibility levels reflect physiological vulnerabilities associated with key growth stages, particularly plant sensitivity to saturated soils, oxygen deprivation in the root zone, delayed development, and reproductive failure. Monthly FSI values reflect documented physiological thresholds for each crop: corn peaks in July (pollination), soybeans in August (pod set/fill), and winter wheat in May (flowering). Agronomic rationale for each crop’s monthly FSI assignments, including evidence on flood tolerance durations by growth stage, is provided in the SI.

Monthly FSI values were assigned using a hybrid framework integrating agronomic literature with phenological evidence, prioritizing reproductive stages over vegetative or dormant periods. Table 3 presents the resulting values.

**Table 3:** Monthly Flood Susceptibility Index (FSI) for corn, soybeans, and winter wheat by phenological stage

Month	Corn	Soybeans	Winter wheat	Rationale / Growth stage
Jan	0.0	0.0	0.1	Dormant season. Corn/soy not planted. Wheat is dormant, very low susceptibility
Feb	0.0	0.0	0.1	Dormant season. Corn/soy not planted. Wheat is dormant.
Mar	0.0	0.0	0.4	Wheat green-up begins. Saturation can promote disease and hinder growth.
Apr	0.7	0.0	0.8	Corn planting; Seedlings are highly vulnerable to ponding. Wheat in stem elongation, highly sensitive.
May	0.9	0.7	1.0	Critical for corn (emergence, early growth). Soybean planting. Wheat flowering - EXTREMELY sensitive.
Jun	0.6	0.5	0.3	Corn in vegetative stage (more resilient). Soybeans are in early vegetative stage. Wheat is in grain filling but nearing harvest.
Jul	1.0	0.6	0.0	CRITICAL - Corn pollination. Maximum sensitivity. Soybeans in mid-vegetative. Wheat harvested, no crop.
Aug	0.8	0.9	0.0	Corn in grain fill (very sensitive to stress). CRITICAL - Soybeans in pod set/fill. Wheat harvested.
Sep	0.3	0.7	0.0	Corn maturing, drying down. Soybeans in seed fill, still sensitive. Wheat harvested, planting soon (low susceptibility)
Oct	0.1	0.2	0.1	Corn and soybean harvest. Minimal yield impact. Winter wheat is being seeded/establishing.
Nov	0.0	0.0	0.1	Post-harvest. Wheat is establishing but slow growing.
Dec	0.0	0.0	0.1	Dormant season. Wheat is dormant.

The FSI values presented in Table 3 represent expert-informed ordinal rankings rather than empirically calibrated damage coefficients. While the relative vulnerability patterns are grounded in agronomic literature on flood tolerance and phenological sensitivity (Bailey-Serres et al. 2012; Timmerman et al. 2018; Pioneer Agronomy Sciences 2026), the specific numerical assignments reflect structured judgment based on: (1) documented survival thresholds for inundation duration at different growth stages, (2) Missouri Extension crop calendars for typical planting and harvest windows, and (3) the principle that reproductive stages exhibit disproportionate vulnerability relative to vegetative or dormant periods. The FSI framework prioritizes internal consistency and transparency over spurious precision. Future refinement could incorporate controlled flooding experiments or econometric estimation from historical loss-event databases, though such data remain limited for U.S. Midwest row crop systems.

### Spatial Data and Flood Extent Characterization

**Fig. 3:**
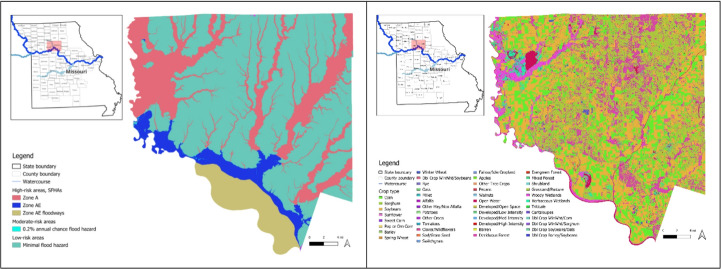
Spatial overlay of FEMA flood hazard zones and 2024 crop distribution in Chariton County, Missouri

**Fig. 4:**
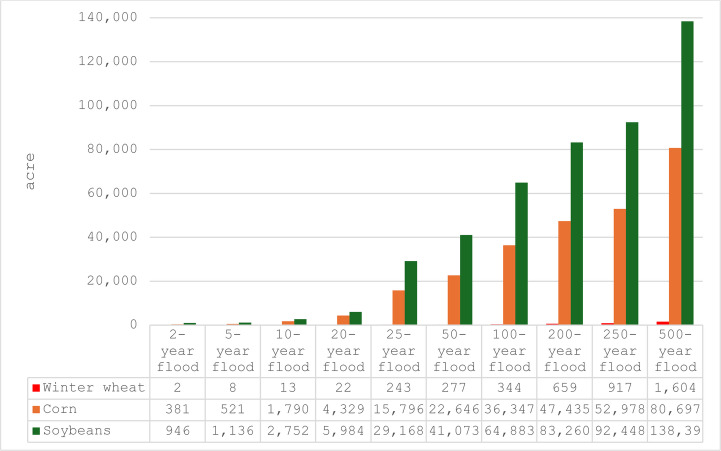
Estimated crop area subject to flooding across return periods, Chariton County

Flood-prone agricultural areas were delineated by overlaying FEMA National Flood Hazard Layer data and USGS Dynamic Surface Water Extent (DSWE) satellite-derived extents with the 2024 USDA Cropland Data Layer (CDL) at 10-m resolution (Fig. 3). For return periods without direct flood mapping (10-, 50-, 200-, 250-year events), inundated crop areas were estimated using power-law scaling relationships fitted to observed FEMA and DSWE extents (R² = 0.91–0.98 across crops; Fig. 4). Full spatial methodology, regression parameters, and cross-validation results are provided in the SI.

### Expected Annual Damage

The final stage of the analysis integrates the flood hazard characterization, crop exposure, and vulnerability information into a unified framework to estimate expected agricultural losses. This step translates flood probabilities, inundation extents, and crop sensitivity into economic impacts using a probabilistic, month- and crop-specific approach.

#### Yield Loss Estimation

For each crop, the per-acre yield loss is calculated as the product of the potential yield, the flood hazard index (FHI), and the crop-specific flood susceptibility index (FSI):

1 $$\:Yield\:Loss=Potential\:Yield\:x\:FHI\:x\:FSI$$

Here, the FHI captures the combined effects of flood intensity and duration, while the FSI reflects crop- and month-specific vulnerability. This formulation assumes that yield reductions increase proportionally with flood severity and crop susceptibility. This proportionality assumption is adopted to ensure transparency and consistency in the absence of detailed empirical damage functions.

#### Financial Loss Calculation

The total financial loss for each crop is obtained by multiplying the yield loss by the market price and the area of the crop exposed to flooding:

2 $$\:Total\:Financial\:Loss=Yield\:Loss\:x\:Crop\:Price\:x\:Flooded\:Area$$

This converts per-acre biological losses into economic terms, accounting for both crop value and spatial exposure.

#### Expected Annual Loss for Each Return Period

To estimate expected losses for a given flood return period, the total financial losses are weighted by the probability that a flood occurs in a particular month and by the annual exceedance probability (AEP) of the event:

3 $$\:{Expected\:Loss}_{RP}=AEP\:x{\textstyle\sum_{Crop}}{\textstyle\sum_{Month}}\left(Total\:Financial\:Loss\:x\:Monthly\:Flood\:Probability\right)$$

This approach captures the probabilistic nature of flood occurrence and ensures that the timing of events within a year is handled correctly, thereby avoiding double-counting.

#### Total Expected Annual Damage

The overall expected annual damage (EAD) is the sum of expected losses across all return periods:

4 $$\:EAD=\sum\limits_{RP}{Expected\:Loss}_{Return\:Period}$$

This represents the long-run average annual loss in economic terms, integrating contributions from floods of varying severity.

#### Seasonal Risk Decomposition

To characterize the temporal distribution of risk, a monthly share of expected loss is calculated for each return period:

5 $$\:Monthly\:Loss\:Share=\frac{\sum_{Crops}\left(Total\:Financial\:Loss\:x\:Monthly\:probability\right)}{\sum_{Crops}\sum_{Month}(Total\:Financial\:Loss\:x\:Monthly\:probability)}$$

This decomposition links hazard occurrence with crop phenology, enabling consistent estimation of potential economic impacts across different flood magnitudes and timing combinations.

## Results

### Overview of Agricultural Flood Risk Profile

The integration of probabilistic flood hazard characterization with spatially explicit crop exposure and phenologically resolved vulnerability functions reveals three structural features of agricultural flood risk in Chariton County, Missouri.

- Expected annual losses concentrate in the 2–50-year return period range; the 2-year and 25-year floods are the largest individual contributors, while events rarer than 200 years contribute only marginally despite generating the largest per-event damages.
- Risk is overwhelmingly associated with the April–July growing season, with a progressive shift toward July dominance as flood magnitude increases; extreme floods are almost entirely confined to mid-summer.
- Corn accounts for the largest share of both gross and probability-weighted losses, followed by soybean; winter wheat contributes only marginally, reflecting both lower floodplain acreage and a growth calendar that partially avoids the peak flood window.
- The expected annual agricultural flood damage is approximately US$7.5 million, representing the long-run average loss under current land use and hydrological conditions.

### Crop-specific Yield Loss Patterns

**Fig. 5:**
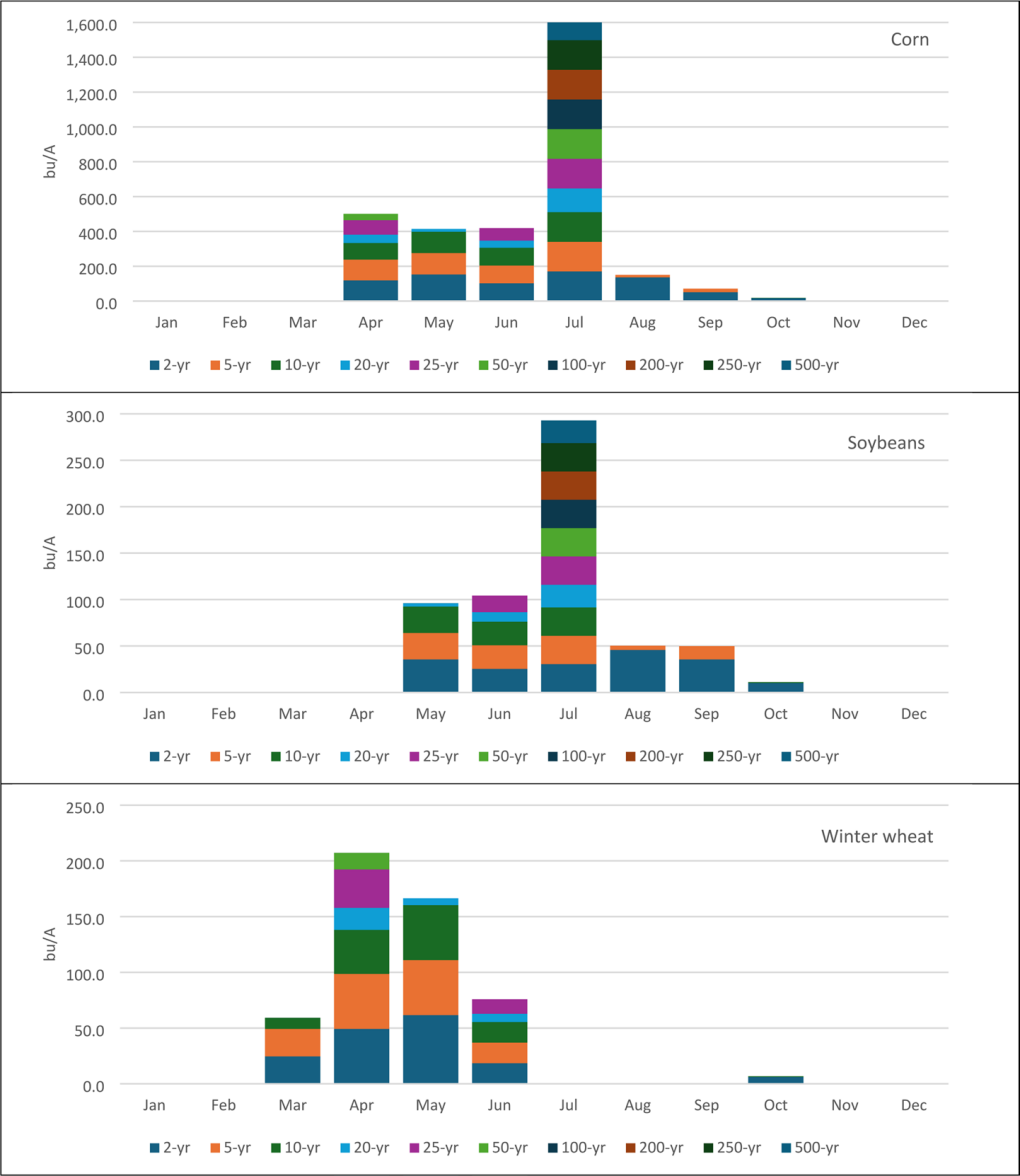
Monthly distribution of conditional crop yield losses by flood return period. Note: Y-axis shows cumulative stacked values — each segment represents yield loss for the corresponding flood return period

Figure 5 presents conditional yield losses by month and return period for all three crops. Corn and soybeans peak in July (pollination and reproduction stages) and winter wheat in April (heading and flowering). Across all crops, losses are highest for frequent to moderate events (2–10-year return periods) and non-monotonic with flood magnitude due to interactions between duration, intensity, and crop stage. Crop-by-crop monthly breakdowns are provided in the SI.

Across all crops, yield losses are non-monotonic with respect to flood return period. Instead, they vary due to interactions among flood magnitude, duration, and crop development stage. Moderate and frequent events (2–10-year return periods) account for the largest and most consistent losses because they more often coincide with sensitive growth periods across the season. In contrast, extreme events (≥ 25-year return periods) may generate limited losses when they occur outside vulnerable stages, although they can produce substantial impacts when alignment occurs.

### Financial Loss Distribution Across Magnitudes and Seasons

**Fig. 6:**
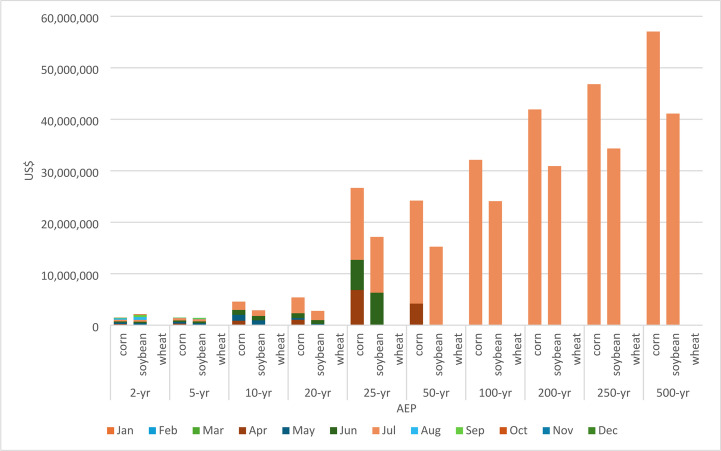
Event-specific agricultural losses by return period and seasonal timing

Figure 6 presents gross agricultural flood losses derived from simulated yield reductions by applying crop-specific prices and the spatial distribution of planted acreage within inundation zones for each flood return period. Reported values are conditional on flood occurrence and therefore represent event-specific economic severity rather than probability-weighted expected losses. Results are disaggregated by crop and by month, allowing assessment of both compositional and seasonal patterns.

Total gross losses increase nonlinearly with flood magnitude. Aggregate damages remain below approximately US$2 million for the 2- and 5-year events and rise modestly to roughly US$4–5 million for the 10- and 20-year floods. A pronounced structural increase occurs at the 25-year return period, where losses rise to approximately US$26–27 million. Beyond this threshold, damages escalate sharply with flood magnitude, reaching about US$24 million for the 50-year event, US$32 million for the 100-year event, US$42 million for the 200-year event, US$46–47 million for the 250-year event, and nearly US$57 million for the 500-year flood. This pattern indicates strong nonlinearity in economic exposure as inundation expands spatially and intersects with higher-value crop stages.

### Comparative Risk Across Return Periods

**Fig. 7:**
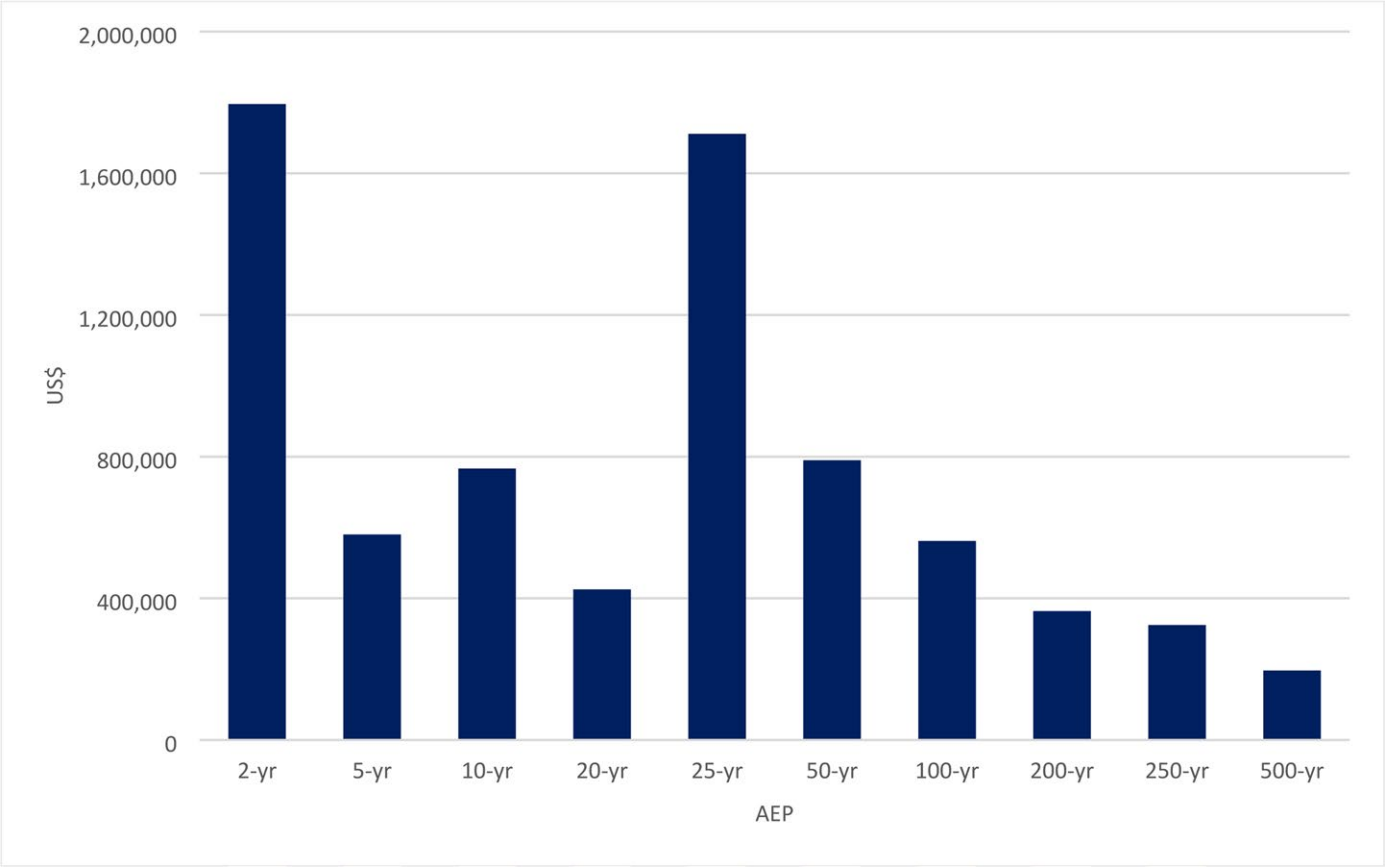
Probability-weighted contribution of each return period to total expected annual agricultural damage, Chariton County

Figure 7 presents probability-weighted agricultural flood losses aggregated across corn, soybean, and winter wheat for each return period. Each bar represents the contribution of a specific flood event to total EAD, calculated as the product of event-specific gross losses and the corresponding AEP. These values therefore reflect expected losses rather than conditional event severity.

The distribution of probability-weighted damages is highly skewed toward frequent flood events. The 2-year flood contributes approximately US$1.8 million to EAD, representing the single largest contribution across all return periods. The 25-year event also contributes substantially (≈ US$1.7 million), reflecting the combination of relatively high event severity and non-negligible probability. In contrast, intermediate return periods such as the 5-, 10-, and 50-year floods contribute between roughly US$0.5–0.8 million each.

Beyond the 100-year return period, contributions decline sharply. The 200-, 250-, and 500-year floods each contribute less than approximately US$0.4 million to EAD, despite their large conditional damages. This sharp decline reflects the rapid reduction in AEP as return periods increase. Although per-event losses rise nonlinearly with flood magnitude (as shown in Fig. 11), the declining probability multiplier dominates in expected-value terms.

Summing across all return periods yields a total expected annual agricultural flood damage on the order of US$7.5 million. This aggregate EAD represents the appropriate economic metric for evaluating agricultural flood risk, as it integrates both event severity and probability. The associated damage–probability relationship provides complementary information by distinguishing the relative importance of frequent, lower-impact floods versus rare, high-impact events, thereby informing the selection of mitigation strategies and acceptable protection standards.

## Discussion

### Interpretation of Risk Patterns

The probabilistic flood damage assessment reveals a risk structure that differs in important ways from conventional assumptions in floodplain management, organized around three interconnected patterns: the dominance of frequent and intermediate floods in expected losses, strong seasonal concentration of risk aligned with crop phenology, and crop-specific vulnerability as a primary driver of aggregate loss distribution.

#### Disproportionate Risk from Frequent and Intermediate Floods

Contrary to the common policy emphasis on rare catastrophic floods, the probability-weighted results show that EAD is concentrated in the 2–50-year return period range, with the 2-year and 25-year events representing the largest individual contributors. Events rarer than 200 years contribute only marginally in expected-value terms despite generating large conditional damages. This pattern follows directly from the structure of expected loss calculation: as return periods increase, exceedance probability declines geometrically while damage increases nonlinearly but not proportionally, such that the declining probability multiplier dominates beyond intermediate return periods. This structure is consistent with established flood risk theory demonstrating that expected losses are often dominated by moderate-frequency events (Merz et al. 2009, 2010), and with evidence that repetitive moderate events frequently generate larger cumulative losses than rare catastrophes (Kunreuther and Michel-Kerjan 2009).

#### Seasonal Concentration and Phenological Alignment

Expected losses are strongly concentrated within the April–July growing season, with an increasing dominance of July as return periods rise. For extreme floods at or above the 100-year level, expected losses are almost entirely concentrated in July. This pattern reflects the synchronization between the regional hydrological regime and crop phenology. In the Lower Missouri Basin, late spring and early summer floods are driven by snowmelt contributions from the upper basin combined with convective precipitation, producing peak discharges that coincide with critical growth stages for corn and soybeans — including vegetative expansion, flowering, and early grain fill — periods documented as highly sensitive to inundation stress (Zaidi et al. 2004; Bailey-Serres et al. 2012). The progressive collapse of the loss distribution into July at higher return periods suggests that extreme floods in this basin are temporally constrained rather than seasonally distributed hazards, and that agricultural flood risk is shaped not only by magnitude and frequency but by the coincidence of hazard timing with peak biological and economic crop value.

#### Crop-Specific Vulnerability, Exposure and Regional Context

Corn accounts for the largest share of both gross and probability-weighted losses, followed by soybean, while winter wheat contributes only marginally to EAD. This distribution reflects three reinforcing mechanisms: greater acreage of corn and soybean within frequently inundated bottomlands, higher per-acre economic value during peak growing months, and greater phenological sensitivity during the dominant flood season. Agricultural flood damage functions exhibit concave behavior beyond physiological tolerance thresholds — once inundation exceeds crop survival duration, additional flood severity yields limited incremental loss — and this biological threshold behavior reinforces the frequency dominance observed in expected-value terms (Bailey-Serres et al. 2012). 

A direct numerical comparison with USACE EAD estimates is not feasible, as project-level outputs for Chariton County are not publicly available. The more fundamental distinction, however, is structural: the USACE NED methodology evaluates damages at seasonal averages and peak discharge stages (USACE 1987), systematically underrepresenting frequent, moderate, long-duration floods that coincide with sensitive crop growth stages. The present framework does not claim superior calibration precision; rather, it resolves a different and complementary risk signal — one that peak-discharge models are architecturally unable to capture regardless of their parameterization.

### Policy and Management Implications

The risk structure revealed by this analysis has several implications for flood risk management in the Lower Missouri Basin. Because 2–50-year floods account for the majority of EAD, policies emphasizing only 100-year or greater protection may address a relatively small share of long-run agricultural risk. Non-structural interventions targeting frequent inundation — such as improved drainage, micro-topographic land shaping, or targeted conservation easements — may yield higher benefit-cost ratios than large-scale structural defenses designed for rare events. This interpretation aligns with FEMA’s recognition that risk reduction strategies should consider the full exceedance probability spectrum rather than single design standards (FEMA 2023).

The strong seasonal concentration of losses suggests that adaptation need not provide year-round protection. NOAA’s Climate Prediction Center issues spring flood outlooks with lead times of weeks to months, offering potential for anticipatory adjustments in planting dates or crop choice. While quantitative estimates of potential loss reduction from forecast-informed adaptation require further modeling, subseasonal-to-seasonal forecast literature suggests meaningful decision value in agricultural contexts (White et al. 2017). Integration of seasonal hydrologic forecasts into farm-level planning could reduce exposure particularly for frequent flood classes.

Crop-specific vulnerability patterns also suggest potential for risk reduction through agronomic adaptation. Flood-tolerant variety research has demonstrated yield preservation under submergence stress in cereals (Bailey-Serres et al. 2012), though commercial adoption in corn and soybeans remains limited. Phenological adjustment through variety selection or modified planting dates represents a plausible adaptation pathway, given that losses concentrate heavily in July at higher return periods — a window that earlier planting or faster-maturing varieties might partially avoid. However, quantifying the realized benefit of such adjustments would require farm-level optimization modeling that accounts for trade-offs between flood exposure and other production risks, including heat stress, shortened growing seasons, and maturity constraints. The analysis therefore identifies agronomic adaptation as a direction warranting further investigation rather than a strategy with quantified benefit-cost support at this stage.

The EAD estimates presented here represent a pre-adaptation baseline — the necessary reference point against which the benefits of any adaptation measure, including crop switching, altered planting dates, or insurance uptake, must be evaluated. The framework directly accommodates such extensions: modifying FSI values for adapted cropping systems or adjusting monthly exposure to reflect alternative planting calendars translates adaptation scenarios into changes in EAD using the same probabilistic structure, without requiring methodological revision.

### Limitations and Uncertainties

Four methodological constraints warrant consideration when interpreting these results.

#### Hydrological Stationarity

Flood frequencies are derived from historical discharge records (1928–2025) and assume stationarity in flood magnitude and seasonal timing. Climate change may alter Missouri River Basin flood regimes through changes in precipitation intensity, snowmelt dynamics, and runoff timing (Milly et al. 2008; USGCRP, 2018). If substantial seasonal shifts occur, the timing and magnitude of expected losses would change accordingly. The present estimates therefore characterize risk under observed historical conditions rather than projected future climates. Extending the framework to incorporate non-stationary flood frequencies derived from downscaled climate projections represents a priority for future work.

#### Flood Extent Estimation

Inundation areas for intermediate return periods (10-, 50-, 200-, 250-year events) are estimated using power-law scaling relationships rather than direct observations. While this approach enables continuous damage estimation across the probability spectrum and is consistent with established hydrological practice, it introduces uncertainty in absolute loss magnitudes. Sensitivity analysis indicates that scaling parameter uncertainty primarily affects EAD totals while preserving relative patterns across return periods and seasons. The availability of high-resolution hydraulic modeling or additional satellite-derived flood observations would reduce this source of uncertainty.

#### Crop Vulnerability Parameterization

FSI and associated damage functions represent structured expert judgment informed by agronomic literature rather than direct econometric calibration. While the ordinal structure—where reproductive stages consistently exceed vegetative sensitivity—is well-supported by experimental evidence (Bailey-Serres et al. 2012; Timmerman et al. 2018), precise cardinal values remain judgment-based. Informal sensitivity testing (± 20% FSI perturbations) suggests that structural findings regarding frequency dominance and seasonal risk concentration are robust to moderate parameter variations, though absolute EAD estimates exhibit corresponding uncertainty. The simplified functional form may understate losses from extreme prolonged inundation (> 15 days) or overstate impacts from brief events (< 2 days), though most historical floods fall within moderate duration ranges where crop physiological responses are better characterized. Field validation using crop insurance claims databases or controlled flooding experiments would improve calibration precision.

#### Scope of Valuation

Estimated damages reflect direct yield losses to standing crops but exclude indirect effects including soil degradation, delayed planting in subsequent seasons, farm infrastructure damage, and market-mediated price adjustments. Indirect losses can materially influence total economic consequences (Merz et al. 2010) and may accumulate over multiple years following severe events. The reported expected annual damage should therefore be interpreted as a lower bound on total agricultural flood impacts. Additionally, estimates represent gross damages before risk transfers through crop insurance or disaster assistance programs; actual farm-level losses would be reduced by insurance penetration rates, though these transfers represent social costs rather than eliminated damages.

Despite these constraints, the analysis provides a transparent and internally consistent probabilistic framework for agricultural flood risk assessment. The methodology integrates hazard probability, spatial exposure, and phenological vulnerability in a reproducible structure that can accommodate improved hydrological projections, empirically calibrated damage functions, or adaptive management scenarios as data become available.

## Conclusion

This study provides a probabilistic, seasonally resolved assessment of agricultural flood risk in Chariton County, Missouri, integrating flood frequency analysis with spatial crop exposure and phenology-sensitive vulnerability functions. By decomposing expected annual damage across return periods, months, and crop types, the analysis moves beyond single-event damage estimation and instead characterizes the structural composition of long-run risk.

The framework is practically accessible without specialized hydrological modeling software. All required inputs — USGS streamflow records, USDA Cropland Data Layers, and FEMA/DSWE flood extents — are freely available and routinely used by state agencies and insurance actuaries. The FHI and FSI are computed from tabular lookup matrices implementable in a spreadsheet; the EAD aggregation involves standard arithmetic. The primary technical step — flood frequency analysis from gauge records — is documented in USACE and USGS guidance and routinely performed by state water resource agencies. For insurance agencies already applying actuarial EAD frameworks, incorporating a month-indexed FSI lookup table in place of a static damage function is an incremental operational modification rather than a system redesign.

While the analysis is grounded in long-term historical records and established frequency estimates, future extensions could incorporate non-stationary flood regimes under climate change, empirically calibrated nonlinear damage functions, and behavioral adaptation responses at the farm level. Expanding the framework to additional river basins would also enable comparative assessment of regional risk structures and adaptation priorities.

## Supplementary Information

Below is the link to the electronic supplementary material.

Supplementary file1 (DOCX 71.7 KB)
