# Supplementary material for: The Cost of Bad Timing: How Phenology and Frequency Determine Agricultural Flood Risk
Source: Water Resour Manag (Dordr). 2026 Jun 15;40(9):417. doi: 10.1007/s11269-026-04801-1 (PMC13269509; doi:10.1007/s11269-026-04801-1)
Supplement: Supplementary file 1 — Supplementary file1 (DOCX 71.7 KB) [file 11269_2026_4801_MOESM1_ESM.docx]

**The Cost of Bad Timing: How Phenology and Frequency Determine Agricultural Flood Risk**

**Supplementary Information**

- - 1. **Temporal Distribution of Flood Probabilities**


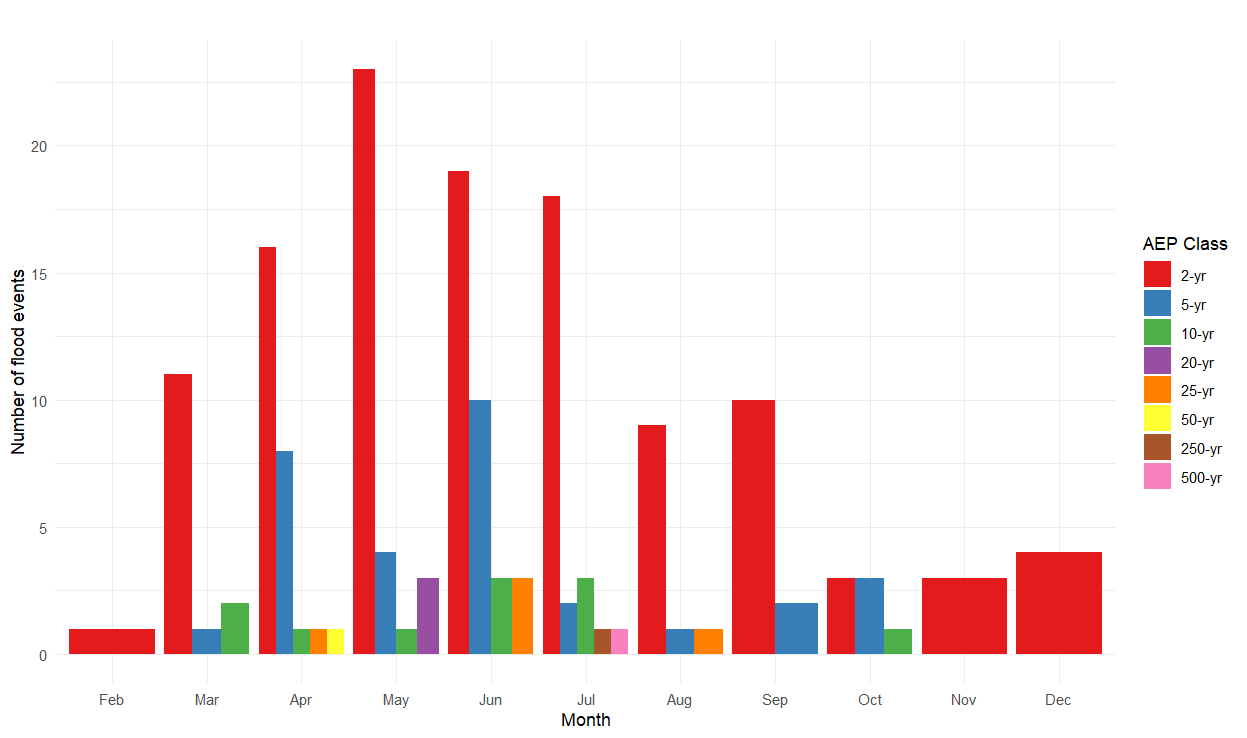


**Fig. 1** Flood frequency by month and return period at Waverly gauge (1928-2025)

Figure 1 presents flood event frequency stratified by both month and AEP class, revealing how seasonal timing varies with flood magnitude. While the overall spring-summer dominance persists across all return periods, notable patterns emerge. The 2-year floods (most frequent events) occur predominantly in March through June with relatively even distribution. More extreme events show increased concentration in specific months—for instance, June experienced 10 events exceeding the 5-year threshold, the highest count for any single month-AEP combination. Notably, extreme floods (≥100-year return period) occurred exclusively during the April-July period in the historical record, suggesting that the largest magnitude floods are temporally constrained to late spring and early summer when snowmelt and precipitation combine to generate peak flows.


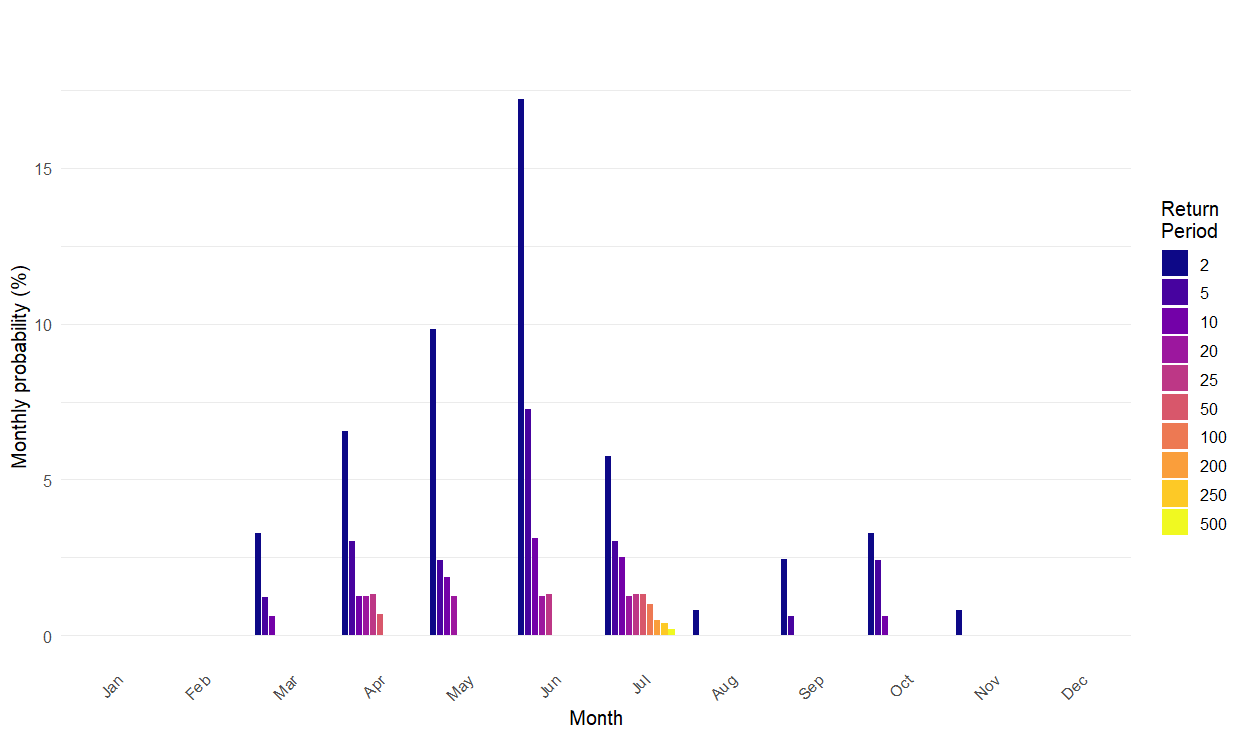


**Fig. 2** Conditional monthly probability of flood occurrence by return period, Waverly gauge, 1928-2025

Figure 2 presents the conditional monthly probability of flood occurrence by return period. Floods of all magnitudes exhibit strong seasonal concentration in late spring to early summer, with extreme events confined exclusively to April–July.

First, floods of all magnitudes exhibit strong seasonal concentration in the late spring to early summer period. The 2-year floods (most frequent events, shown in dark blue) display the broadest seasonal distribution, occurring primarily from March through October with peak probability in June (18% of events). As flood magnitude increases, the seasonal distribution becomes progressively more concentrated. The 5-year floods (purple) show 73% of events occurring during the April-July period, while 10-year floods (lighter purple) exhibit 83% occurrence during these same four months.

Second, extreme magnitude floods (≥50-year return period) demonstrate marked temporal constraint, occurring exclusively during the April-July window in the historical record. The 100-year floods (orange) and rarer events (yellow, pink) show complete absence outside this period, indicating that the hydrological conditions necessary to generate extreme floods—likely the combination of upper basin snowmelt and intense precipitation—occur only during late spring and early summer. This temporal constraint has important implications for agricultural risk assessment, as it limits the phenological stages during which crops face exposure to catastrophic flooding.

Third, a secondary but notable pattern emerges in the fall months (September-October), where 2-year and 5-year floods show modest probability (3-4% each month). These autumn floods, while less frequent and lower in magnitude, coincide with critical harvest periods and warrant consideration in comprehensive risk assessment.

- - 1. **Flood Duration Analysis**

The duration thresholds are consistent with experimental and extension-based evidence on flood tolerance for row crops (Fig. 3) (Timmerman et al. 2018; Shen et al. 2023; Pioneer Agronomy Sciences 2026). These categories, established from experimental studies of corn, soybeans, wheat, and sorghum flood tolerance, enable translation of continuous duration values into discrete damage states corresponding to vulnerability functions in the crop damage model. Empirical studies show that soybean yields are largely unaffected by flooding of ≤3 days but decline sharply with longer inundation, with severe yield reductions at 6–9 days of flooding depending on growth stage. Corn and soybean survival also declines rapidly with flood durations beyond 4 days, reflecting increasing physiological stress, root impairment, and reduced water and nutrient uptake with prolonged soil saturation (Shen et al. 2023). Corn at early vegetative stages (e.g., V1–V6) can often withstand ~2–4 days of saturated soils, while soybeans typically survive ~48 hours but show *significant stand and yield losses* with ≥4 days of flooding and more severe impacts with prolonged inundation (Pioneer Agronomy Sciences 2026).

The use of discrete rather than continuous duration classes reflects both data constraints and agronomic reality. Empirically calibrated continuous duration-damage functions for Midwest row crops are not available at the resolution needed to justify their use; moreover, crop flood tolerance operates through biological thresholds — not smooth gradients — making categorical classes agronomically more appropriate than interpolated continuous functions. Most historical flood events at Waverly fall clearly within a single duration category, minimizing boundary misclassification. As shown in the sensitivity analysis (Section 4.3), structural findings on frequency dominance and seasonal risk concentration are robust to the specific thresholds applied.


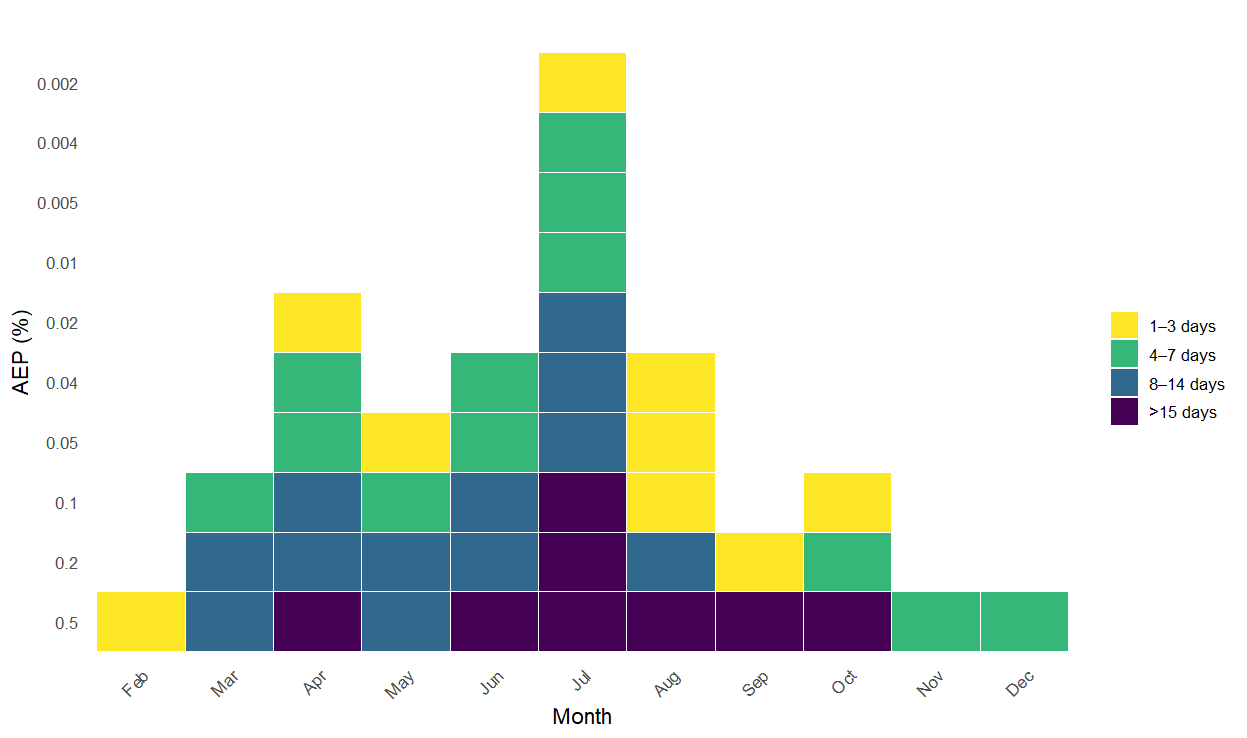


**Fig. 3** Flood duration patterns by month and return period, categorized by agronomic damage thresholds

- - 1. **Flood Hazard Index (FHI)**

The index is developed using a discrete hazard matrix in which flood duration classes (1–3, 4–7, 8–14, and ≥15 days) are jointly evaluated with four intensity levels (low, medium, high, and very high). Each duration–intensity combination is assigned an FHI value ranging from 0.1 to 1.0, reflecting an increasing likelihood and comparative severity of crop stress or damage. The FHI is an ordinal severity index rather than an empirically calibrated damage function and is used to preserve relative differences in flood hazard across timing, intensity, and duration (Table 2).

By construction, the FHI increases monotonically with both duration and intensity, reaching unity for prolonged floods (≥15 days) and for shorter events occurring under very high intensity conditions. This formulation allows flood hazards to be translated into a consistent relative severity metric that can be temporally aligned with crop growth stage calendars and stage-specific vulnerability functions. As a result, the framework explicitly accounts for the timing, magnitude, and persistence of flooding, recognizing that agricultural flood risk emerges from their combined influence rather than from peak discharge alone.

- - 1. **Flood Susceptibility Index (FSI)**
- Corn exhibits its highest susceptibility during pollination, typically occurring in July, which is a critical yield-determining stage. Susceptibility is also elevated during early vegetative development and grain filling. During early growth stages, particularly prior to the V5–V6 stage when the growing point remains near or below the soil surface, corn tolerance to saturated soils is limited, with survival often restricted to approximately two to four days of complete soil saturation (Thomison 1995; Pioneer Agronomy Sciences 2026). Flooding beyond this duration can severely impair plant development and yield formation.
- Soybeans generally demonstrate greater tolerance to temporary flooding than corn, particularly during vegetative growth. Yield losses are often minimal when flooding persists for less than 48 hours; however, extended flooding progressively reduces plant vigor and yield potential. Flood durations exceeding four days commonly delay plant growth and reduce plant height and node development, while six or more days of flooding can substantially reduce yields and potentially destroy plant stands (Naeve 2004; Timmerman et al. 2018). Susceptibility increases markedly during reproductive stages, especially from flowering through pod set and filling (R1–R5 stages), when flooding has been shown to cause measurable daily yield losses and disrupt nitrogen fixation processes (Scott et al., 1989; Pioneer Agronomy Sciences 2026). Accordingly, maximum susceptibility is assigned during August, when pod development and seed filling dominate crop growth.
- Winter wheat susceptibility differs due to its overwintering growth cycle. The crop exhibits low vulnerability during winter dormancy and following harvest, while susceptibility increases during spring green-up and stem elongation and reaches its peak during flowering, typically occurring in May. Although small grains can tolerate short-term flooding, often surviving one to two days of saturated soil conditions, prolonged inundation during reproductive stages significantly reduces yield formation (Peel 2000; Berglund 2005). Compared with corn, wheat generally exhibits slightly higher tolerance to short-duration flooding but remains vulnerable during critical reproductive phases.

Across crops, growth stage strongly influences flood survivability because plant oxygen requirements and submergence risks vary throughout development. Early growth stages are often highly vulnerable because young plants are more easily submerged and physiologically stressed by saturated soils, while larger plants require greater oxygen supply to sustain metabolic processes (Pioneer Agronomy Sciences 2026). These biological dynamics justify assigning temporal variability to susceptibility rather than assuming uniform vulnerability throughout the growing season.

Monthly FSI values were therefore assigned using a hybrid framework that integrates literature evidence with synthetic evaluation of dominant crop growth stages. For corn and soybeans, susceptibility is assumed to be zero during winter months when crops are absent, increases during planting and early growth, peaks during reproductive development, and declines toward maturity and harvest. Peak susceptibility is assigned to corn during pollination (July) and to soybeans during pod set and seed filling (August). Winter wheat exhibits low susceptibility during dormancy, increasing vulnerability during spring growth and flowering, followed by a rapid decline after harvest.

The FSI does not represent direct yield loss estimates or probabilistic damage functions. Instead, it provides a structured and internally consistent representation of relative crop vulnerability that can be integrated with the Flood Hazard Index and monthly flood probabilities to quantify seasonal flood risk. This formulation enables flood damages to vary not only with hydrologic severity but also with crop-specific biological timing, which is fundamental for realistic agricultural flood damage assessment.

## **Spatial Data and Flood Extent Characterization**

The spatial component of the analysis integrates FEMA flood hazard mapping and remote sensing-derived water extent with high-resolution cropland data (USDA NASS, 2024) to delineate flood-prone agricultural areas in Chariton County, Missouri. Flood hazard information was obtained from FEMA's National Flood Hazard Layer (NFHL), which provides digital floodplain maps at the county scale (<https://www.fema.gov/flood-maps/national-flood-hazard-layer>). Chariton County has an effective FEMA flood hazard map; therefore, its full spatial extent was included in the analysis. Flood hazard zones were extracted from FEMA's Flood Map Service Center (https://msc.fema.gov/) and grouped into three aggregated risk categories consistent with FEMA classifications. High-risk areas correspond to Special Flood Hazard Areas (SFHAs), including Zones A, AE, AH, AO, and designated AE floodways, representing areas subject to the 1% annual chance flood. Moderate-risk areas include Shaded Zone X, which captures areas subject to the 0.2% annual chance flood, shallow flooding, or areas protected by levees. Low-risk areas include Unshaded Zone X and Zone D, representing areas outside the 0.2% annual chance flood or where flood hazards are possible but not well defined (Fig. 3).

FEMA provides flood maps only for 1% and 0.2% annual exceedance probability (AEP) events. To characterize more frequent floods, we employed the USGS Dynamic Surface Water Extent (DSWE) product. The DSWE method delineates surface water using surface reflectance from Landsat and Moderate Resolution Imaging Spectroradiometer (MODIS) satellite imagery combined with terrain and hillshade data. DSWE classifies water pixels into six categories (not water, high-confidence water, moderate-confidence water, partial surface water, low-confidence water, and snow/cloud) based on multiple spectral indices and bitwise logical tests (EROS 2022). High- and moderate-confidence water pixels were interpreted as flood extents in this study. Due to imagery availability and cloud contamination constraints, DSWE-derived surface water extents for Chariton County were obtained for the 50%, 20%, 5%, and 4% AEP events, following the latest USGS implementation (Walker et al. 2025). To ensure consistency between the FEMA and DSWE flood maps, we assumed that any area that remained dry in a more severe flood event would also remain dry in the map for a less severe flood event.

Agricultural land exposure within flood hazard zones was quantified using the 2024 Cropland Data Layer (CDL) produced by USDA's National Agricultural Statistics Service (https://www.nass.usda.gov/Research_and_Science/Cropland/Release/). The CDL provides crop-specific land cover at 10-m spatial resolution. FEMA and DSWE flood zones were rasterized to match CDL resolution using nearest-neighbor resampling, then intersected with the CDL to estimate the area of major crops (corn, soybeans, and winter wheat) within each flood risk category. All spatial overlays and area calculations were conducted at the county scale, ensuring consistency between flood hazard delineation and agricultural exposure estimates (Fig. 3).

This spatial framework provides county-specific flood exposure estimates that integrate directly with flood hazard intensity, duration metrics, and crop susceptibility indices to quantify agricultural flood risk in Chariton County.

Figure 4 illustrates estimated crop areas potentially affected by flooding across a range of annual exceedance probabilities for winter wheat, corn, and soybeans. Flooded areas for the 1% and 0.2% AEP events are based on direct spatial overlay of FEMA flood hazard maps with the Cropland Data Layer, representing empirically observed exposure. For more frequent events (2-, 5-, 10-, 20-, 25-, and 50-year floods), FEMA does not provide mapped extents; flooded crop areas at these return periods were estimated using power-law scaling relationships.

Flood inundation typically expands nonlinearly as flood magnitude increases because larger flood events involve increased water depth, floodplain connectivity, and storage capacity. Therefore, flooded crop acreage $A$was modeled as a power function of flood return period $T$(years):

| $A(T)=\alpha T^{\beta}$ | (2) |
| --- | --- |

where:

$A$= flooded crop area (acres)

$T$ = flood return period (years)

$\alpha=$scale factor

$\beta$ = elasticity parameter describing the rate at which flooded area increases with flood magnitude

Taking natural logarithms transforms the model into a linear regression form:

| $\ln(A)=a+ \beta ln( T)$ | (3) |
| --- | --- |

where $\alpha=ln(\alpha)$

Separate regressions were estimated for winter wheat, corn, and soybeans using six observed flood return periods (n = 6):

- Remote sensing–derived flood extents for 2-, 5-, 20-, and 25-year events
- FEMA floodplain estimates for 100- and 500-year events

Predicted return periods (10-, 50-, 200-, and 250-year floods) were not included in model estimation and were generated only after model calibration.

Ordinary Least Squares (OLS) regression was applied to log-transformed data. Model performance was evaluated using coefficient of determination (R²), adjusted R², parameter significance tests, confidence intervals, and residual diagnostics. Due to the limited sample size, model stability was evaluated using leave-one-out cross-validation (LOOCV) rather than data splitting.

Flooded areas for the 10-, 50-, 200-, and 250-year events represent statistical interpolations and extrapolations derived from the estimated power-law relationships and were not directly observed. Prediction uncertainty increases with return period, particularly beyond the 100-year event.

**Table 1** Power-law regression results for flooded crop areas

| **Crop** | **Intercept (**$\boldsymbol{\alpha}$**)** | $\boldsymbol{\beta(Elasticity)}$ | **95% CI (**$\boldsymbol{\beta}$**)** | $\boldsymbol{R}^{\boldsymbol{2}}$ | **Adj.** $\boldsymbol{R}^{\boldsymbol{2}}$ | **p-value** | **n** |
| --- | --- | --- | --- | --- | --- | --- | --- |
| Corn | 4.528 | 0.972 | [0.74, 1.20] | 0.97 | 0.96 | <0.001 | 6 |
| Soybeans | 5.379 | 0.915 | [0.71. 1.12] | 0.98 | 0.97 | <0.001 | 6 |
| Winter wheat | -0.896 | 1.202 | [0.68, 1.73] | 0.91 | 0.89 | 0.003 | 6 |

Estimated elasticities (β) range from 0.915 to 1.202, indicating approximately proportional to slightly super-proportional expansion of inundated cropland with increasing flood return period. These values exceed typical hydraulic floodplain width scaling (0.3–0.6) reported in geomorphic studies because the dependent variable represents cultivated land area rather than physical channel width. Agricultural fields are spatially clustered in low-elevation floodplain zones; once flood depth exceeds topographic thresholds, large contiguous cropland areas become inundated, generating nonlinear expansion patterns. Leave-one-out cross-validation produced mean absolute percentage errors of 18.4% for winter wheat, 9.2% for corn, and 7.8% for soybeans, indicating moderate predictive stability despite the limited sample size.

## **Crop-specific Yield Loss Patterns**

For corn, July consistently represents the period of maximum vulnerability across nearly all flood return periods, coinciding with pollination and early grain-filling stages. During this month, expected yield losses remained high and relatively stable, reaching approximately 170 bu/A for most return periods, with slightly lower losses (approximately 136 bu/A) observed for the 20-year and 500-year events. This pattern likely reflects differences in flood duration and timing within the month. Moderate vulnerability is observed during spring, particularly from April through June, although losses vary substantially across return periods. In April and May, yield losses are highest for more frequent flood events, reaching approximately 119–153 bu/A for 2-year floods, but generally decline as flood return periods increase. In June, losses remain elevated for frequent to moderate floods (approximately 102 bu/A for 2- to 10-year events) but become negligible for larger return periods. Vulnerability declines sharply after July. While August still shows measurable losses for more frequent floods (approximately 136 bu/A for 2-year events), susceptibility decreases markedly for less frequent events and becomes minimal by September. Yield losses are negligible from October onward, reflecting crop maturity and harvest completion.

Soybeans exhibit a comparable seasonal vulnerability pattern to corn but with substantially lower yield loss magnitude. Peak vulnerability occurs during mid-summer, particularly in July, corresponding to key reproductive growth stages. During this month, expected yield losses remain relatively consistent at approximately 30.5 bu/A across most return periods, with slightly lower losses (approximately 24.4 bu/A) observed for the 20-year and 500-year flood events. Moderate vulnerability is also evident during May and June, when losses are highest for more frequent flood events. In May, yield losses reach approximately 35.6 bu/A for 2-year floods but decline sharply for less frequent events. Similarly, June losses remain moderate for frequent floods (approximately 25.4 bu/A for 2- to 10-year events) and diminish with increasing return periods. August represents a transitional period, with substantial losses observed only for frequent flooding (approximately 45.8 bu/A for 2-year events), followed by a rapid decline in vulnerability for less frequent floods. By September, losses are limited primarily to frequent flood events, while yield impacts become negligible by October and remain absent throughout the post-harvest period. Overall, soybean flood vulnerability is concentrated within the late vegetative and reproductive growth stages and is strongly influenced by flood frequency and seasonal timing.

Winter wheat exhibits an earlier vulnerability window than summer crops due to its overwintering growth cycle. Peak vulnerability occurs during spring development, particularly in April and May, corresponding to stem elongation, heading, and flowering stages. In April, expected yield losses range from approximately 49.3 bu/A for 2- and 5-year flood events to about 14.8 bu/A for 50-year floods, with negligible impacts for larger return periods. May represents the period of maximum losses, with expected yield reductions reaching approximately 61.7 bu/A for 2-year floods and remaining substantial for 5- and 10-year events, while declining sharply for less frequent floods. Moderate vulnerability is observed in March and June, when losses reach approximately 24.7 bu/A and 18.5 bu/A, respectively, for frequent flood events but diminish for higher return periods. Yield impacts are negligible during mid- and late summer months, reflecting crop maturity and harvest completion. Minor losses observed in October and November are associated with early establishment stages following fall planting. Overall, winter wheat flood vulnerability is concentrated in spring reproductive and grain development stages and is primarily driven by frequent to moderately frequent flood events.
